# A systematic review on the association of metabolic syndrome with telomere length and telomerase activity

**DOI:** 10.6026/973206300213047

**Published:** 2025-09-30

**Authors:** Ipsita Dash, Bibhu Prasad Behera, Gaurav Gupta, Om Padarabinda Dash, Pratima Kumari Sahu

**Affiliations:** 1 Department of Biochemistry, Sahed Rendo Majhi Medical College, Kalahandi, Odisha, India; 2 Department of Internal Medicine, Saheed Rendo Majhi Medical College, Kalahandi, Odisha, India; 3 Department of Pediatrics, All India Institute of Medical Sciences, Guwahati, Assam, India; 4 Department of Pulmonary Medicine, Sahed Rendo Majhi Medical College, Kalahandi, Odisha, India

**Keywords:** Metabolic syndrome, telomere length, telomerase activity, cellular aging, oxidative stress, biomarkers

## Abstract

Metabolic syndrome (MetS) is a cluster of conditions that increases the risk of cardiovascular disease and type 2 diabetes and has been linked to accelerate cellular aging. MetS participants exhibited shorter telomeres and disrupted telomerase activity, suggesting increased biological aging. Lifestyle interventions, particularly high-intensity interval training (HIIT) and mindfulness practices, were associated with telomere elongation and improved telomerase activity, whereas pharmacological approaches showed inconsistent results. Thus, we show the protective role of lifestyle modification, large-scale randomized controlled trials across diverse populations are needed to validate long-term benefits on cellular aging and disease outcomes.

## Background:

Metabolic syndrome (MetS) is a constellation of interconnected conditions that is reflected through abdominal obesity, hypertension, hyperglycemia, and dyslipidemia [1]. MetS is associated with a significant increased risk for cardiovascular disease and type 2 diabetes [2]. One predominant pathological mechanism that interrelates these conditions is increased oxidative stress, which leads to decreased insulin sensitivity, chronic inflammatory processes, and endothelial dysfunction. Together, these conditions result in accelerated metabolic decline while adding to cellular aging [3]. Telomeres, which are protective caps at the ends of chromosomes, are known to shorten with age. In addition to chronological aging, oxidative stress adds to this telomere attrition. Telomerase is an enzyme that maintains telomere length to facilitate cellular repair and longevity. Reduced telomerase function in conjunction with telomere shortening is increasingly considered a biological marker of aging and disease progression in metabolic syndrome [4]. According to evidence, oxidative stress in MetS hastens telomere shortening, while also inhibiting telomerase activity, leading to a cycle of metabolic dysregulation and cellular senescence [5]. Decreased telomerase activity, was previously demonstrated in patients suffering from obesity and diabetes, and is statistically related to higher cardiovascular risk and mortality [6]. Interventions that reduce oxidative stress and increase telomerase activity, in either lifestyle changes or pharmacological interventions, may provide novel opportunities to slowdown the progression of MetS, and prevent its sequelae. Therefore, it is of interest to systematically review the current evidence on the associations between metabolic syndrome, telomere length, and telomerase activity.

## Materials and Methods:

A comprehensive literature search was conducted using PubMed, Scopus, and Web of Science, to identify studies published in English between January 2000 and December 2024, using keywords such as "metabolic syndrome," "telomere," "telomere length," "telomerase activity," and "oxidative stress." Studies were included if they involved adults with metabolic syndrome or related conditions and assessed the impact of lifestyle, pharmacological, or other interventions on telomere length or telomerase activity, using suitable comparators and quantitative outcome measures. Randomized controlled trials, cohort, cross-sectional, and case-control studies were eligible. Animal studies, in vitro experiments, studies lacking primary outcome data, or with poor data quality, and non-original research articles were excluded. Two independent reviewers screened studies as per PRISMA guidelines, resolving disagreements through consensus or a third reviewer. Data were independently extracted using a standardized form, capturing study details, participant characteristics, intervention types, measurement methods, baseline and post-intervention values, statistical outcomes, and indicators of risk of bias and methodological quality.

## Results:

This systematic review included 11 studies presented in Table 1 (see PDF): having study references [7, 8, 9, 10, 11, 12, 13, 14, 15, 16- 17]. The study populations were diverse, including individuals diagnosed with MetS, healthy adults, and those with related conditions such as obesity, insulin resistance, and cardiovascular disease. Most studies focused on MetS patients, while a few targeted healthy individuals undergoing lifestyle interventions or patients with acute coronary syndrome. Participant ages ranged from 30 to 70 years, with both sexes represented, though ethnicity was infrequently reported. Interventions were categorized as exercise-based (*e.g.*, high-intensity interval training, resistance training), lifestyle-based (*e.g.*, mindfulness, diet), pharmacological (*e.g.*, telomerase activators) and observational.

Table 1 (see PDF) presents the characteristics of all 11 studies included in the systematic review. It summarizes the study population; type of intervention or study design, outcomes measured, and key findings. Studies involving exercise-based interventions, such as LV-HIIT, resistance training, and WB-EMS, reported positive effects on telomere length and cardiometabolic health. Studies focusing on mindfulness and intensive meditation interventions showed a significant increase in telomerase activity. One study assessing a pharmacological intervention (TA-65MD) reported no significant effect on telomere length. Observational and cohort studies reinforced the finding that individuals with MetS exhibit shorter telomere length compared to controls; further supporting the hypothesis that metabolic dysfunction accelerates cellular aging. Additionally, lifestyle interventions combining diet, exercise, and stress management were associated with improvements in both telomere length and telomerase activity. Collectively, the table highlights that non-pharmacological lifestyle modifications yield more consistent and favorable outcomes in modulating telomere biology in individuals with or at risk for MetS.

## Discussion:

Current data shows a consistent association between MetS and markers of cellular aging, specifically, shortened telomere length and altered telomerase activity. The rstudies collectively indicate a potential link between lifestyle interventions and telomere biology in individuals with metabolic risk factors. Exercise, particularly low-volume high-intensity interval training (LV-HIIT), consistently demonstrated beneficial effects on telomere length (TL), suggesting it may play a protective role in cellular aging [7, 8, 13]. Other exercise modalities such as resistance training (RT), whole-body electromyostimulation (WB-EMS), and endurance training showed varied but generally favorable impacts on telomere dynamics [13]. These findings underscore the importance of physical activity in modulating biological aging processes among those with MetS, [18] although the magnitude and consistency of the effects appear to differ based on the type of intervention. In contrast, pharmacological approaches such as TA-65MD did not produce significant changes in TL, highlighting the limitations of current anti-aging supplements in altering telomere biology [9]. On the other hand, psychosocial interventions like mindfulness and intensive meditation demonstrated a notable increase in telomerase activity, a key enzyme involved in telomere maintenance [19]. Additionally, comprehensive lifestyle interventions encompassing diet, exercise, and stress reduction also contributed to both preservation of TL and enhancement of telomerase activity. Observational and cohort studies further reinforced that individuals with MetS tend to have shorter TL, strengthening the hypothesis of a negative association between metabolic dysfunction and cellular aging [20]. The review highlights several research gaps, including a lack of randomized controlled trials directly comparing MetS patients with healthy controls, limited ethnic diversity, and inconsistent telomere assessment methods. Additionally, mechanistic pathways linking MetS and telomere biology remain underexplored, particularly in interventional contexts. Although the biological rationale for lifestyle benefits is strong, the long-term effects of such interventions on telomere dynamics and age-related disease outcomes are yet to be clarified. Future studies should prioritize large, multi-ethnic RCTs with standardized methods, investigate dose-response effects of interventions, and explore synergistic pharmacological-lifestyle strategies.

## Conclusion:

MetS is linked to accelerate cellular aging, marked by shorter telomeres and altered telomerase activity. Exercise and lifestyle interventions, especially HIIT and mindfulness, improved telomere biology, while pharmacological approaches were less effective. Future multi-ethnic trials are needed to confirm these findings and explore long-term benefits.

## Financial support:

No Funding
